# Diet-Induced Proteomic and Metabolomic Signatures in Chronic Kidney Disease: A Precision Nutrition Approach

**DOI:** 10.3390/metabo15030211

**Published:** 2025-03-20

**Authors:** Sandra Cabała, Agnieszka Herosimczyk

**Affiliations:** Department of Physiology, Cytobiology and Proteomics, Faculty of Biotechnology and Animal Husbandry, West Pomeranian University of Technology Szczecin, Klemensa Janickiego 29, 71-270 Szczecin, Poland; sandra.cabala@zut.edu.pl

**Keywords:** kidney, urine, plasma, metabolome, proteome, nutrition, chronic kidney disease

## Abstract

**Background:** Diet is a key modifiable factor that can either support renal health or accelerate the onset and progression of chronic kidney disease (CKD). Recent advances in multiomics, particularly proteomics and metabolomics, significantly enhanced our understanding of the molecular mechanisms linking diet to CKD risk. Proteomics offers a comprehensive analysis of protein expression, structure, and interactions, revealing how dietary components regulate cellular processes and signaling pathways. Meanwhile, metabolomics provides a detailed profile of low-molecular-weight compounds, including endogenous metabolites and diet-derived molecules, offering insights into the metabolic states that influence kidney function. **Methods:** We have conducted a narrative review of key papers from databases such as PubMed, Scopus, and Web of Science to explore the potential of proteomic and metabolomic analysis in identifying molecular signatures associated with diet in human and animal biological samples, such as blood plasma, urine, and in kidney tissues. These signatures help elucidate how specific foods, food groups, and overall dietary patterns may either contribute to or mitigate CKD risk. **Results:** Recent studies the impact of high-fat diets on protein expression involved in energy metabolism, inflammation, and fibrosis, identifying early biomarkers of kidney injury. Metabolic, including disruptions in in fatty acid metabolism, glucose regulation, and amino acid pathways, have been recognized as key indicators of CKD risk. Additionally, several studies explore specific metabolites found in biological fluids and renal tissue in response to protein-rich foods, assessing their potential roles in a progressive loss of kidney function. Emerging evidence also suggests that dietary interventions targeting the gut microbiota may help alleviate inflammation, oxidative stress, and toxin accumulation in chronic kidney disease. Notably, recent findings highlight metabolomic signatures linked to beneficial shifts in gut microbial metabolism, particularly in the context of prebiotic supplementation. **Conclusions:** By integrating proteomics and metabolomics, future research can refine precision nutrition strategies, helping mitigate CKD progression. Expanding large-scale studies and clinical trials will be essential in translating these molecular insights into actionable dietary guidelines.

## 1. Introduction

The kidneys play a crucial role in maintaining homeostasis by filtering metabolic waste, regulating fluid balance, and producing hormones essential for blood pressure and red blood cell production [1,2,3]. In addition to waste removal, the kidneys also maintain long term acid–base balance and they play crucial endocrine roles by secreting various hormones and humoral factors [4]. This unique ability to manage the internal biochemical environment underscores the importance of kidney function in sustaining homeostasis and overall health.

Diet is a well-known, modifiable factor that can either promote renal health [5,6] or accelerate the onset and progression of kidney disease [7,8,9]. In a recent paper, Van Westing et al. [10] analyzed the relationships between specific foods such as red (processed) meat, poultry, fish, dairy, legumes, nuts, and fruits, as well as beverages including coffee, tea, sugar-sweetened beverages (SSBs), and diet beverages, on the incidence of chronic kidney disease (CKD) in adults. In this context, the authors [10] also assessed the effects of various dietary interventions, such as dietary approach to stop hypertension (DASH) diet, Mediterranean diet, high-fat and high-sugar diets, as well as diets with a high acid load with incidence of CKD across various cohort studies. Van Westing et al. [10] presented compelling evidence suggesting that plant-based foods, coffee, and dairy product may reduce the risk of CKD, whereas high-fat and high-sugar diets, along with components such as red meat and SSBs, may contribute to kidney function decline. Joshi et al. [11], in their recent review, reached a similar conclusion, emphasizing that a diet rich in unprocessed, whole, plant-based foods may offer significant benefits for patients with CKD.

Nowadays, integrating multiple -omics techniques emerged as an innovative and comprehensive approach, offering deeper insights into the molecular and physiological mechanisms underlying the roles of nutrients and other dietary factors in maintaining overall health or contributing to chronic diseases such as CKD [12,13]. Among these high-throughput techniques, proteomics and metabolomics emerged as particularly valuable, as proteins and metabolites are dynamic and highly modifiable by diet, making them ideal candidates for therapeutic targeting. The proteome and metabolome reflect the cumulative outcomes of gene function and are therefore often grouped under the broader discipline of “functional genomics”, which seeks to elucidate the genome-wide connections between genotype and phenotype [14].

Proteomics focuses on the complete evaluation of proteins, including their expression levels, structures, functions, and interactions [15]. This is particularly significant as nutritional processes depend on the precise coordination of a vast array of proteins that are synthesized and secreted at the cellular level. It is important to emphasize that these processes can be influenced by various factors, including the overall composition of the diet, the levels of specific macro- and micro-nutrients, and other lifestyle factors [16]. Food consists of a variety of complex components that are processed in the gastrointestinal tract (GIT) to release and absorb nutrients. These nutrients and their metabolites serve not only as essential building blocks for cellular structures and energy production, but also act as signaling molecules [17]. By binding to specific receptors, they activate transcription factors, which, in turn, regulate the transcription of targeted genes. This process leads to alterations in gene expression patterns and subsequent protein synthesis, influencing various cellular functions and biological processes [18]. It should also be noted that the dietary components can influence protein expression levels and induce post-translational modifications (PTMs), which are covalent modifications occurring on one or more amino acids after protein translation. These PTMs, including glycosylation, phosphorylation, ubiquitination, acylation, and methylation, regulate protein activity, localization, folding, as well as interactions with other small molecules. Since most catalytic and regulatory pathways function as interconnected networks, with extensive cross-talk between various signaling pathways, even a minor change in the structure or activity of a single protein can have far-reaching consequences. Such alterations may disrupt signaling cascades, influence the metabolic process, or modify cellular responses, ultimately leading to significant physiological or pathological effects in an individual [18,19]. Recently, Lobel et al. [20] provided a missing element in this equation, demonstrating that diet can also induce post-translational modifications in the gut microbial proteome. Using a mouse model of CKD, they found that a diet rich in sulfur-containing amino acids led to modifications of microbial tryptophanase activity. Specifically, their study revealed that sulfide, produced through bacterial metabolism of dietary sulfur amino acids, regulates *E. coli* indole production by inhibiting tryptophanase via S-sulfhydration. These findings highlight how dietary components can be metabolized by the microbiota to induce post-translational modifications in microbial proteins, ultimately influencing host health and providing a framework for understanding host–diet–microbiota interactions in disease states such as CKD [20].

**Figure 1:**
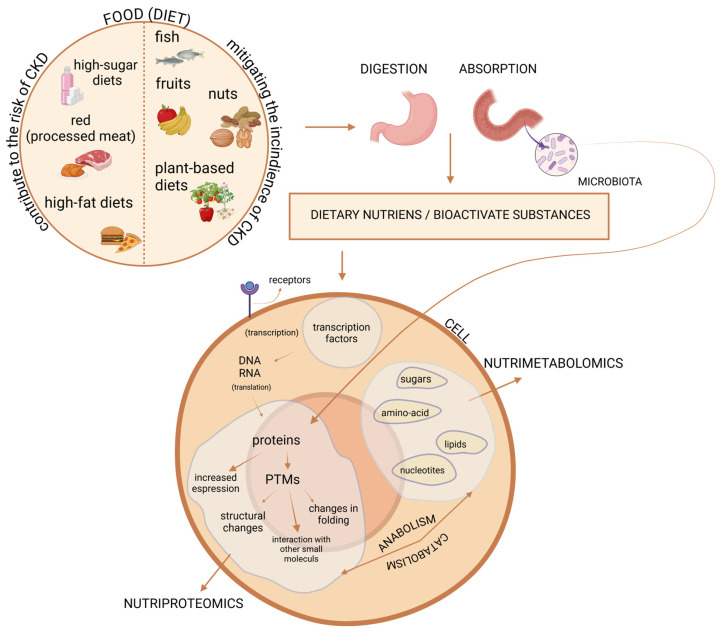
Integration of proteomics and metabolomics in understanding diet-induced molecular changes. Food is composed of diverse complex components that are digested in the gastrointestinal tract to extract nutrients. These nutrients and their metabolites act as signaling molecules, influencing gene expression and protein synthesis. They can also directly induce post-translational modification (PTMs), altering protein activity, localization, folding, and interactions with other molecules. Additionally, gut microbiota metabolizes dietary compounds, further driving PTMs. As the final products of cellular process, metabolites provide crucial insights into metabolic state. The integration of proteomics and metabolomics into nutrition research—nutriproteomics and nutrimetabolomics—uncovers molecular signatures that link diet to kidney function. Created in BioRender software version 04. Cabała, S. [2025] https://BioRender.com/n52z682 (accessed on 27 February 2025).

Metabolomics represents the final stage of omics analysis, focusing on low-molecular-weight compounds such as amino acids, lipids, and organic acids. These metabolites, as end products of cellular processes, provide insights into systemic metabolic states [21]. As outlined by Guasch-Ferré [22], diet can impact two distinct components of the metabolome. The first is the endogenous one, which includes all metabolites naturally present in a biological sample from the host. The second is the food metabolome, comprising metabolites derived from ingested food and processed by the organism. It should be highlighted that many of them are then filtered and excreted by the kidneys, offering valuable insights into the relationship between diet, metabolites, and kidney function [23]. Recently, these two powerful techniques have been integrated into the field of nutrition, giving rise to the disciplines of nutriproteomics and nutrimetabolomics. As integral branches of proteomics and metabolomics, they seek to identify and quantify the effects of dietary intervention on protein expression and metabolite production level—collectively referred to as the “molecular signatures” (Figure 1) [18,24]. Therefore, nutriproteomics employs proteomics-based methods to examine how whole diets, specific foods, or selected bioactive compounds influence protein alterations across various tissues and biological fluids [18]. In contrast, nutrimetabolomics provides a similar opportunity but focuses on analyzing components of the metabolome [21].

As nutriproteomics and nutrimetabolomics are still emerging fields, the existing literature is limited and does not cover all dietary interventions. For our literature review, we used the following search terms: “chronic kidney disease”, “proteomics”, “metabolomics”, “high-fat diet”, “protein-rich foods”, and “pre-, pro- and synbiotics”. We searched relevant articles in the databases PubMed, Scopus, and Web of Science. Given this constraint, our review highlights the potential of proteomic and metabolomic analysis in identifying molecular signatures found in human and animal biological samples, such as blood plasma, urine, and in some cases, kidney tissues. These signatures are linked to the intake of specific foods and food groups, as well as overall dietary patterns, which may either contribute to or mitigate the risk of diet-related CKD.

## 2. Molecular Signatures Associated with the Intake of Protein-Rich Foods and Their Connection to CKD

Although no formal definition of a high-protein diet was established, it is generally described by most authors as a protein consumption ranging from 1.3 to 2.0 g/kg per day [25,26]. Excessive protein ingestion (when protein constitutes > 35% of total energy intake), is known to induce renal hypertrophy, glomerular hyperfiltration, elevated renal plasma flow, as well as proteinuria. This maladaptive response may contribute to the progression of kidney diseases [25,26,27]. Results from both human and animal studies, including interventional and observational research, suggest a potential link between dietary protein intake and chronic kidney disease (CKD) progression. However, as reviewed by Ko et al. [25], some findings remain inconsistent, highlighting the need for further analysis. One proposed mechanism for high-protein diet-induced hyperfiltration is its role in facilitating the excretion of increased protein-derived nitrogenous waste [25]. A study by Sällström et al. [28] suggests that this phenomenon occurs independently of the tubuloglomerular feedback mechanism (TGF) and nitric oxide synthases, contrary to previous assumptions [25]. Instead, the authors postulate that the glomerular growth increases the filtration surface area, directly contributing to the elevated glomerular filtration rate (GFR). Furthermore, vascular endothelial growth factor may play a crucial role in glomerular hypertrophy and hyperfiltration [28].

Dietary proteins can be broadly classified as either animal-based or plant-based. It is well established that animal-sourced proteins, particularly those found in eggs, milk, and fish offer higher nutritional values compared to plant proteins. This advantage is primarily due to their complete amino acid profiles and superior digestibility [29]. In contrast, plant-based proteins are less digestible, partly due to their fiber content and the presence of antinutritional factors. Moreover, plant proteins often lack a sufficient amount of essential amino acids, such as leucine, sulfur amino acids, or lysine, which means that their amino acids are more likely to be oxidized rather than used for muscle protein synthesis [30]. Nonetheless, several observational studies, as comprehensively reviewed by Molina et al. [31], suggest that plant-based proteins may confer certain renal benefits. These include reducing proteinuria, lowering uremic toxins levels, decreasing phosphorus intake, and reducing acid production. These effects could potentially slow CKD progression, although the exact underlying mechanisms remain largely unknown. To address this knowledge gap, Bernard et al. [23] employed an untargeted metabolomics approach to investigate the relationships between the consumption of various protein-rich foods (fish, nuts, legumes, red and processed meat, eggs, and poultry) and the serum metabolites produced. Their main goal was to link these metabolic changes to the incidence of chronic kidney disease in middle-aged adults. Interestingly, their study revealed a renoprotective association with a metabolite derived from animal-based dietary proteins rather than plant-based sources. Specifically, they found that 1-docosahexaenoylglycerophosphocholine (22:6n3), a fish-related metabolite belonging to the glycerophosphocholine (GPC) lipid species, was linked to a lower risk of CKD. A similar study by Ren et al. [32] investigated children with diagnosed CKD to analyze changes in plasma metabolites associated with a higher dietary intake of different protein-rich foods, including red and processed meat, chicken, fish, eggs, nuts, and beans. Using nontargeted metabolomics analysis, the researchers found contradictory findings. On one hand, the plasma lipid 1-(1-enyl-palmitoyl)-2-oleoyl-glycerophosphoethanolamine (GPE, P-16:0/18:1) was positively associated with red and processed meat intake and strongly linked to an increased risk of CKD progression—doubling its levels corresponded to an 88% higher risk. On the other hand, 3-ureidopropionate, a nucleotide, showed an inverse relationship with red and processed meat consumption, with its doubling linked to a 48% lower risk of CKD progression [32]. While these findings provide intriguing insights into how various dietary proteins may impact kidney health, further research is needed to fully understand their significance.

As mentioned earlier, there is no universal consensus on the optimal diet for reducing kidney disease risk or the impact of protein source and intake levels in CKD patients. Clinical guidelines recommend limiting protein intake to >1.3 g/kg/day for those at risk of CKD progression and reducing it to 0.8 g/kg/day for patients with severely decreased GFR (<30 mL/min/1.73 m^2^) [33]. To explore this further, Rebholz et al. [33] conducted a metabolomic study to identify serum metabolites associated with different levels of dietary protein intake from both animal and plant sources. The study included two groups based on CKD progression: participants with moderate CKD (GFR: 25–55 mL/min/1.73 m^2^) were randomly assigned to a low- or moderate-protein diet, while those with severe CKD (GFR: 13–24 mL/min/1.73 m^2^) followed either a very low or low-protein diet with keto acid for 12 months. The findings highlight the significant impact of dietary protein levels on essential amino acid pathways and protein metabolism markers. Across both studies, notable differences were observed, particularly in histidine-related compounds, branched-chain amino acids (BCAAa), and creatinine, which emerged as reliable indicators of animal protein intake [33]. Additionally, 11 metabolites were consistently linked to protein consumption in both studies, reinforcing their potential as dietary biomarkers. Notably, 3-methylhistidine, creatine, carnitine derivatives, and trimethylamine N-oxide (TMAO) were strongly associated with protein intake, whereas lipid-related metabolites increased with lower protein consumption, suggesting shifts in macronutrients balance. Furthermore, the study [33] identified novel metabolites, such as kynurenate and xanthurenate, which are involved in tryptophan metabolism. These findings not only validate known biomarkers of protein intake, but also reveal new metabolic pathways that could be crucial for CKD management and nutritional research. The results further support the use of blood-based metabolomic profiling as a valuable tool for dietary assessment and disease monitoring.

Among protein-rich foods, sheep milk stands out not only as a valuable source of protein, but also as a rich reservoir of bioactive compounds including fatty acids, immunoglobulins, hormones, vitamins, and minerals that offer antibacterial, antiviral, and anti-inflammatory benefits [34]. These properties make sheep milk a promising candidate for slowing the progression of CKD. Recent research by Wei et al. [35] utilized proteomic and metabolomic analyses in an adenine-induced CKD murine model to explore these renoprotective effects. Considering the differences in basal metabolic rates between humans and mice, the authors adjusted the equivalent dose of sheep milk to align with human consumption standards, based on body weight and the milk’s dry matter content. For four weeks, mice were fed either a control diet or a diet containing 0.2% adenine supplemented with 1.25 mL of sheep milk daily. The study demonstrated that sheep milk exerts significant nephroprotective effects by modulating key metabolic and signaling pathways in renal tissue. Specifically, Wei et al. [35] showed that sheep milk improved protein, lipid and mineral absorption, while enhancing hormonal metabolism and alleviating oxidative stress, inflammation, and fibrosis. Sheep milk supplementation led to a marked reductions in renal mRNA levels of pro-inflammatory markers, including intercellular adhesion molecule 1 (ICAM-1), vascular cell adhesion protein 1 (VCAM-1), IL-6, and tumor necrosis factor alpha (TNF-α). Additionally, a significant increase in antioxidant enzymes, including superoxide dismutase (SOD), glutathione peroxidase (GSH), and catalase (CAT) was observed in murine blood plasma. According to the authors, these effects are likely attributable to the milk’s rich content of antioxidants, lactoferrin, and essential trace elements such as zinc. Proteomic analysis of renal tissue revealed decreased expression of fibrosis-associated proteins, including VCAM1, which is typically induced by pro-inflammatory factors in the vascular endothelium and facilitates immune cell adhesion. Since VCAM1 plays a critical role in kidney disease associated with renal fibrosis and chronic inflammation, its down-regulation suggests the protective effects of sheep milk. Additionally, reductions in various collagen types, key components of the extracellular matrix, indicate a potential role in slowing CKD progression. Metabolomic profiling further demonstrated beneficial shifts in renal metabolites, particularly a reduction in TMAO, a known biomarker of kidney damage linked to inflammation, fibrosis, oxidative stress, and thrombosis via activation of the NLR family pyrin domain containing 3 (NLRP3) inflammasome and nuclear factor-κB (NF-κB) signaling. By integrating the proteomic and metabolomic data, Wei et al. [35] showed the down-regulation of the JAK1/STAT3/HIF-1α signaling pathway, with significant reduction in key components, including Janus kinase 1 (JAK1), signal transducer and activator of transcription 3 (STAT3), collagen type I alpha 2 chain (COL1A2), fibronectin 1 (FN1), VCAM1, and ICAM1. These findings collectively support the potential of sheep milk as a nephroprotective agent in kidney disease. The study’s comprehensive analytical approach not only strengthens the credibility of its findings, but also provides new insights into the modulation of key metabolic and signaling pathways, particularly the JAK1/STAT3/HIF-1α axis [35]. By integrating nutriproteomics and nutrimetabolomics with nephrology research, this study represents a significant contribution to understanding the molecular mechanisms underlying the protective effects of dietary interventions in CKD. Table 1 summarizes the studies discussed, presenting additional technical data and highlighting key findings on the relationship between protein-rich food intake and CKD.

## 3. Molecular Signatures Associated with the Intake of High-Fat Diets and Their Connection to CKD

A high-fat diet (HFD) is characterized by a significant intake of both saturated and unsaturated fats, typically making up 30–60% of total energy intake. Excessive dietary fat consumption is strongly associated with obesity and systemic metabolic disorders such as diabetes, hypertension, ischemic heart disease, and steatohepatitis [36]. This is partly due to expansion of adipose tissue, which is known to secrete various immune-modulatory proteins. Obesity promotes increased expression of pro-inflammatory adipokines and decreased expression of anti-inflammatory ones [37]. This low-grade chronic inflammatory state contributes to the development of insulin resistance (IR), which is further exacerbated by the increased lipolysis of adipose tissue [38]. During this process, free fatty acids (FFAs) are released into the bloodstream and transported to peripheral tissues, including the kidneys, triggering lipotoxicity that in turn activates inflammatory, fibrogenic, and apoptotic pathways, ultimately resulting in irreversible cell damage and renal dysfunction [39,40,41,42,43]. Mitochondrial impairment recently emerged as a key factor in this process, with increased reactive oxygen species (ROS) production closely linked to chronic kidney disease progression [44]. Mitochondria, essential for energy production, are highly dynamic organelles that continuously undergo fusion and fission in response to metabolic and environmental stresses, such as excessive fat intake. Fusion helps mitigate cellular stress by mixing contents of partially damaged mitochondria, while fission not only generates new mitochondria, but also removes damaged ones, thereby facilitating apoptosis under severe stress [44,45]. Recent work by Sun et al. [45] provides compelling evidence that mitochondrial dysfunctions play a key role in renal impairment in C57BL/6 mice subjected to long-term HFD feeding. Beyond the typical metabolic disturbances associated with HFD, including obesity, diabetes, elevated FFAs, pro-inflammatory cytokines, and abnormal lipid deposition in the kidneys and liver, the study also reported increased renal oxidative stress. Notably, enhanced mitochondrial fission was linked to cytochrome c release, activation of apoptotic pathways, and excessive renal cell apoptosis. Histological analysis further revealed significant alterations, such as glomerular fibrosis, podocyte foot process effacement, and tubular cell apoptosis [45]. Moreover, in vitro studies confirmed that exposing HK-2 tubular or mesangial cells to high concentrations of glucose, fatty acids, and tumor necrosis factor-alpha (TNF-α) exacerbates these pathological processes. However, inhibiting ROS was found to mitigate cellar injury [45]. Given the growing body of knowledge on the mechanisms underlying obesity-induced chronic kidney disease (CKD), many aspects remain unexplored. Recent advances in high-throughput techniques, particularly proteomics and metabolomics, expanded our understanding of these dietary influences at the molecular level and further clarify the pathways involved in disease progression. In this context, Dozio et al. [46] analyzed the kidney proteome in mice and identified significant protein expression changes in response to a high-fat (HF) diet. Their bioinformatics analysis revealed that the animals fed a HF diet exhibited up-regulation of 96 and down-regulation of 37 proteins compared to the control group. These proteins were associated with key cellular components, including mitochondria, the endoplasmic reticulum, the plasma membrane, and the extracellular matrix. Proteins with reduced expression were associated with thermogenesis and peroxisomal FFA beta-oxidation via acyl-CoA oxidase, while those with increased expression played roles in short-chain fatty acid metabolism, the citric acid cycle (TCA) cycle, oxidative phosphorylation, and mitochondrial translocation, suggesting a metabolic shift from peroxisomal to mitochondrial fatty acid oxidation. The study also revealed a decreased expression of enzymes involved in amino acids catabolism. Additionally, early signs of kidney damage were linked to altered membrane protein expression, with the MYC proto-oncogene emerging as a key transcriptional regulator [46]. These findings suggest that several identified proteins could serve as early biomarkers of HF diet-induced kidney damage, preceding histomorphological changes typically seen in obesity-related CDK. Integrating these proteomic insights with biofluid analysis may enhance early detection and prevention of kidney injury in clinical practice. A similar study by Wypych et al. [47] investigated the effects of a three-month HF diet with varying fatty acid compositions on oxidative stress markers and the mouse kidney proteome. Mice were fed HF diets enriched with either saturated fatty acids (SFA diet) or the HFDs with polyunsaturated fatty acids at a linoleic to α-linolenic acid ratio of 14:1 (HR diet) or 5:1 (LR diet). Proteomic analyses showed that the SFA diet up-regulated acyl-CoA thioesterase 2, D-lactate dehydrogenase, and apolipoprotein E. All HF diets reduced expression of mitochondrial ATP synthase F1 beta subunit, while only the LR diet increased the expression of ETHE1 persulfide dioxygenase and electron transfer flavoprotein (A subunit), suggesting enhanced fatty acid oxidation. Additionally, the SFA and HR diets down-regulated proteins involved in cellular protection while up-regulating inflammatory and apoptotic regulators, such as peptidyl isomerase A. The study highlighted that diets high in saturated fats significantly alter the expression of proteins critical for energy metabolism. These changes disrupt mitochondrial function, ATP synthesis, and fatty acid oxidation, suggesting that excessive saturated fat intake impairs the kidney’s ability to regulate energy production efficiently. Moreover, the findings confirm that HF diets not only disrupted energy metabolism, but also induce oxidative stress in the kidneys, reinforcing the link between HFD consumption and CKD progression. Oxidative stress results from an imbalance between ROS production and the body’s antioxidant defenses, leading to damage to proteins, lipids, and DNA, ultimately contributing to kidney dysfunctions. The authors [47] postulate that these molecular alterations could serve as early indicators of kidney damage, underscoring the long-term risks associated with HF diets.

Alongside the HFD, the Western diet (WD) has been also recognized as a key driver of the global obesity epidemic. Characterized by high-fat, predominantly saturated, high-sugar, and low-fiber foods, the WD includes energy-dense foods such as red and processed meats, as well as refined grains [48]. Beyond its role in obesity, the WD may also have direct adverse effects on kidney function. In this context, Oe et al. [49] recently investigated the impact of the WD on the kidney proteome in C57BL/6J mice to explore its potential role in CKD progression. In their study, mice were fed a WD for eight weeks, leading to significant changes in kidney protein expression compared to the control group. A total of 19 proteins were altered, with 12 predominantly expressed in the proximal tubules, six of which were up-regulated and six down-regulated. According to Oe et al. [49], these changes suggest WD-induced stress in the proximal tubules and the kidney’s adaptative responses. Notably, the authors observed increased expression of argininase 2 (Arg2) and carboxylesterase 1d (Ces1d), which play protective roles by mitigating renal lipid accumulation and preventing tubular injury. Conversely, the down-regulation of 11β-hydroxysteroid dehydrogenase type 1 (Hsd11b1) may further reduce lipid accumulation. Additionally, the down-expression of aminocarboxymuconate semialdehyde decarboxylase (Acmsd) could enhance NAD+ production, potentially safeguarding the kidney tubules against acute stress. Metabolic adaptations were also evident, as indicated by the WD-induced down-regulation of glutamate ammonia ligase (Glul) and up-regulation of glutamate dehydrogenase 1 (Glud1) and phosphoenolpyruvate carboxykinase 1 (Pck1), which suggest enhanced ammoniagenesis and gluconeogenesis. These processes may contribute to bicarbonate formation, providing protection against metabolic acidosis. The most strongly up-regulated protein was glucuronosyltransferase (Ugt2b37), located in the inner mitochondrial membrane, which is predicted to facilitate the conjugation and elimination of toxic xenobiotics. Furthermore, the WD reduced the expression of phosphoglycerate dehydrogenase, an enzyme involved in the early steps of L-serine synthesis. A down-regulation of this protein has been associated with lower circulating serine levels, which may contribute to renal lipid accumulation. Furthermore, these authors also investigated the effect of a HFD in a murine model of Balkan nephropathy, a condition induced by aristolochic acid (AA). AA is taken up by proximal tubular cells via basolateral organic anion transporters 1 and 3 (OAT1/3), leading to DNA damage, which in turn triggers a DNA damage response and acute tubular injury. Their findings demonstrate that the WD feeding exacerbates disease progression in this model, likely by increasing the basolateral uptake of organic anions, including AA, by proximal tubular cells [49].

**Figure 2:**
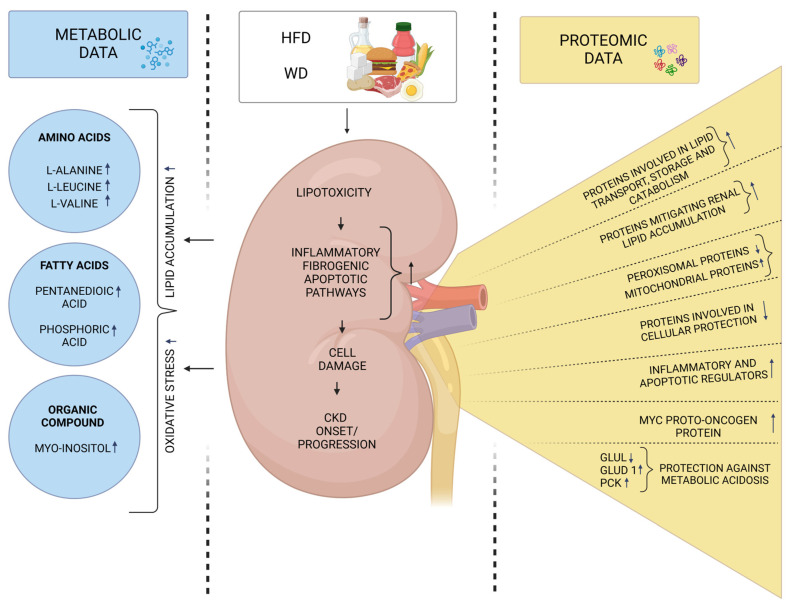
Summary of protein and metabolite changes in renal tissue induced by a high-fat diet (HFD) and Western diet (WD). Excessive dietary fat intake promotes lipotoxicity in the kidneys by increasing the release of free fatty acids (FFAs), which activate inflammatory, fibrogenic, and apoptotic pathways, ultimately leading to renal dysfunction. Recent findings further confirm these mechanisms, reinforcing the role of impaired mitochondrial dynamics, altered protein expression, and metabolite imbalances in renal tissue. Glul—glutamine synthetase, Glud1—glutamate dehydrogenase 1; and PCK—phosphoenolpyruvate carboxykinase Up arrows (↑): Indicate increased protein expression or metabolite concentration. Down arrows (↓) Represent decresed protein expression or metabolite concentration Created in BioRender software version 04. Cabała, S. [2025] https://BioRender.com/q61m670 (accessed on 27 February 2025).

Another key objective is to comprehensively understand the functional significance of various metabolic alterations that may potentially contribute to the risk of HF diet-related CKD. While research on this topic remains limited, a recent study by Xie et al. [50] reported that long-term HFD feeding led to moderate metabolomic changes in the renal tissue of C57BL/6 J mice. Six metabolites were up-regulated in the kidneys, including three two fatty acids (pentanedioic acid, phosphoric acid), and one organic compound (myo-inositol). These metabolic shifts further reinforce the well-established link between high-fat diet intake and kidney injury, likely driven by lipid accumulation and increased oxidative stress in renal tissue. As previously mentioned, insulin resistance (IR) is highly prevalent in individuals with renal dysfunctions and is often characterized by hyperglycemia, glucose intolerance, hyperinsulinemia, and dyslipidemia. Excessive dietary fat intake is also a major contributor to IR [51]. Expanding on this, Xu et al. [51] used a metabolomics approach to compare metabolic alterations across serum, liver, and muscle tissues between CKD-induced and HFD-induced IR rats. Their findings revealed significant differences in metabolic pathways between these two models. Notably, CKD-IR rats exhibited pronounced disruptions in tryptophan and arginine metabolism, suggesting that declining renal function plays a key role in metabolic disturbances. Furthermore, some altered metabolites exhibited opposite trends in CKD-IR and HFD-IR models, highlighting distinct molecular mechanisms underlying IR in these conditions. The study also underscored the role of uremic retention molecules (URMs) and amino acid dysregulation in CKD-induced IR, whereas HFD-IR was primarily linked to disruptions in glucose homeostasis [51]. Table 2 summarizes the studies discussed, presenting additional technical data and highlighting key findings on the relationship between HFD and WD intake and CKD progression. Figure 2 summarizes the findings from the selected discussed studies on protein and metabolite changes in renal tissue induced by a high-fat diet (HFD) and Western diet (WD).

## 4. Molecular Signatures Associated with the Intake of Pre-, Pro-, and Synbiotics and Their Connection to CKD

The gut microbiota is essential for maintaining overall health, protecting against pathogens, and regulating immune system function. Among the various factors shaping its composition, diet plays a crucial role. In particular, non-digestible carbohydrates, known as prebiotics, such as inulin, fructooligosaccharides, and galactooligosaccharides, serve as a primary energy source for beneficial gut bacteria. Through fermentation, these dietary compounds are converted into short-chain fatty acids (SCFAs), including lactic acid, butyric acid, and propionic acid, which support gut health and contribute to metabolic regulation. Consequently, their effects extend beyond the gastrointestinal tracts (GIT), influencing distant organs such as the kidney [52]. Probiotics, on the other hand, are live microorganisms that, when consumed in adequate amounts, help balance the gut microbiota by increasing beneficial bacteria in the GIT. Found in fermented foods and supplements, probiotics support digestive health, enhance immune function, and compete with harmful bacteria. Strains from the *Lactobacillus*, *Streptococcus*, and *Bifidobacterium* genera play a crucial role in maintaining microbial equilibrium, reducing inflammation and limiting the production of harmful metabolites such as uremic toxins [53]. Synbiotics combine prebiotics and probiotics to maximize their benefits, improving probiotic survival and activity in the gut. By fostering a favorable gut environment, synbiotics enhance nutrient absorption, improve gut barrier function, and modulate immune responses [54]. Emerging research suggests that dietary supplantation with prebiotics, probiotics, and synbiotics may hold therapeutic potential in managing kidney diseases by alleviating inflammation, oxidative stress, and toxin accumulation. A meta-analysis by Firouzi and Haghighatdoost [55] found that these dietary interventions significantly reduce blood urea and blood urea nitrogen (BUN) levels in individuals with impaired renal function. However, their analysis did not find statistically significant effects on glomerular filtration rate (GFR) or creatinine levels. Additionally, a study by Rossi et al. [56] highlights that synbiotics can effectively lower protein-derived uremic toxins, such as p-cresyl sulfate (PCS) and indoxyl sulfate (IS), compounds that are difficult to eliminate through dialysis and may also pose significant risk factors for high cardiovascular mortality in patients with chronic kidney disease (CKD). PCS and IS are products of bacterial amino acid fermentation in the large intestine, and their production is exacerbated by CKD-related mechanisms, such as increased urea diffusion, prolonged intestinal transit time, and gut microbiota imbalances. The combination of increased production and impaired clearance leads to toxin accumulation in the bloodstream, which correlates with disease severity [57]. Modulating gut microbiota and intestinal transit time presents a promising therapeutic strategy for reducing the production of these toxins [58]. Prebiotics and probiotics may help create a more favorable gut environment by lowering pH, increasing the production of SCFAs, and inhibiting the bacterial enzymes responsible for PCS and IS synthesis. Lowering these toxins may not only support kidney health, but also enhance BUN excretion [56,59].

The current literature on proteomic and metabolomic analyses of these aforementioned bioactive dietary compounds and their potential role in mitigating CKD risk remains highly limited (Table 3). Most studies focused exclusively on prebiotic supplementation, highlighting a significant gap that warrants further investigation. In a notable study, Zybailov et al. [60] used comparative metaproteomics to analyze the cecal contents of CKD rats fed a diet containing resistant starch (RS) with those fed a diet containing digestible starch (DS). Their analysis identified 179 host proteins with differential abundance between the CKD-DS and CKD-RS groups. Specifically, 125 proteins were down-regulated in the RS-fed rats, approximately half of which were enzymes, while the remaining were associated with the humoral immune response and epithelial–mesenchymal transition. In contrast, 54 proteins were up-expressed in the RS group, including a subset in which one-third were enzymes. The most prominent functional groups included immunoglobulins and annexins—proteins that serve as intracellular Ca^2+^ sensors and play a role in membrane repair. Additional proteins related to voltage-dependent ion channels and sodium pump subunits were also identified, with their down-regulation potentially contributing to increased oxidative stress in CKD. Overall, the study demonstrated that RS supplementation not only attenuates CKD progression, but also induces a significant shift in the gut microbial ecosystem. An increased abundance of butyrate-producing bacteria, along with a reduction in mucin-degrading bacteria, suggest that gut bacteria derived butyrate may help alleviate oxidative stress and inflammation. Furthermore, the authors found that by reducing mucin degradations, RS may enhance the integrity of the gut epithelial barrier, thereby limiting the translocation of harmful metabolites into the bloodstream [60].

Sohn et al. [61] also conducted a study to assess the safety and efficacy of oligofructose-enriched inulin (p-inulin) in modulating the gut microbiome and its metabolic outputs in CKD patients. To evaluate the systemic effects of gut-derived metabolites, the researchers performed comprehensive metabolomic analyses on plasma and urine samples. Their findings reveal an increased urinary excretion of several carbohydrate metabolism products, including raffinose, 1-kestose, and beta-gentiobiose. Raffinose, a non-digestible trisaccharide, is metabolized by gut microbes into gases such as hydrogen, carbon dioxide, and methane that promote the growth of beneficial bacteria such as *Bifidobacteria* [61]. Similary, 1-kestose supports the proliferation of butyrate-producing bacteria, particularly *Faecalibacterium prausnitzii*. Additional, beta-sitosterol, a phytosterol with immunomodulatory, anti-inflammatory, and lipid-lowering effects was enriched in the urine during the dietary intervention. In the post-intervention phase, the study reported an increase in urinary 4-methylcatechol, a flavonoid metabolite derived from rutin, known for its antioxidant and antihypertensive effects. Interestingly, the authors found an inverse correlation between 4-methylcatechol and p-cresol, suggesting a competitive dynamic in their production. Overall, the metabolic changes observed by Sohn et al. [61] appear to result from prebiotic metabolism, shifts in microbial composition, and alterations in microbial metabolic pathways influenced by substrate availability during and after treatment. This study thus underscores the potential of p-inulin to beneficially modulate the gut microbiome and its metabolic products in CKD patients, paving the way for further clinical investigations.

## 5. Conclusions

Current research on diet-induced proteomic and metabolomic signatures and their potential role in chronic kidney disease (CKD) onset and progression remains limited, highlighting the urgent need for further investigation. While proteomics and metabolomics offer powerful tools for uncovering the molecular mechanism, the vast amount of data generated can be both insightful and, at times, challenging to interpret. Despite these complexities, several novel biomarkers associated with dietary protein intake and CKD risk have been identified. These include kynurenate and xanthurenate, both linked to tryptophan metabolism. Additionally, the fish-derived metabolite 1-docosahexaenoylglycerophosphocholine (22:6n3) has been associated with a lower risk of CKD progression, suggesting a protective role for certain animal protein sources. Other potential protective biomarkers include 3-ureidopropionate, a nucleotide inversely associated with CKD risk, and urinary 4-methylcatechol, which has been linked to beneficial shifts in gut microbial metabolism in studies on prebiotic supplementation. Regarding high-fat diet (HFD)-related CKD progression, recent studies reinforce well-established mechanisms, further validating the role of impaired mitochondrial dynamics and altered protein expression in renal tissue. However, whether these biomarkers will be integrated into routine clinical assessment remains an open question. By integrating proteomics and metabolomics, future research can refine precision nutrition strategies, helping mitigate CKD progression. Expanding large-scale studies and clinical trials will be essential in translating these molecular insights into actionable dietary guidelines.

## Figures and Tables

**Table 1:** Summary of molecular signatures identified to date that are associated with protein-rich food intake and their potential links to chronic kidney disease (CKD).

Sample Type	Study Design	Type of Analysis/Analysis Platform	Major Findings	Clinical Implications	Reference
Serum	A prospective cohort study of 3726 middle-aged participants with atherosclerosis risk in communities without CKD at baseline. Study examined the impact of six protein-rich foods (fish, nuts, legumes, red and processed meat, eggs, and poultry) on serum metabolites over 1 year. Associations were analyzed using multivariable linear regression and meta-analyzed with fixed-effects models, adjusting for key demographic and lifestyle factors.	Untargeted metabolomic analysis/ GC-MS LC-MS	Thirty significant associations were found between protein-rich foods and serum metabolites (fish, *n* = 8; nuts, *n* = 5; legumes, *n* = 0; red and processed meat, *n* = 5; eggs, *n* = 3; and poultry, *n* = 9). Metabolites improved the discrimination of high protein intake beyond covariates. Fish consumption was positively linked to 1-docosahexaenoylglycerophosphocholine (22:6n3), which was inversely associated with incident CKD.	These findings support using metabolomic markers to improve dietary assessment and kidney disease prevention. The link between fish consumption, 1-docosahexaenoylglycerophosphocholine (22:6n3), and lower CKD risk reinforces guidelines recommending a Mediterranean diet for CKD patients.	Bernard et al. [23]
Plasma	A prospective cohort study of 484 cases of chronic kidney disease in children participants. Linear regression examined associations between dietary intake of total, animal, and plant protein, as well as chicken, dairy, nuts and beans, red and processed meat, fish, and eggs. Cox models assessed the link between protein-related metabolites and CKD progression, adjusting for demographic and clinical covariates.	Untargeted metabolomic analysis/ LC-MS	Sixty metabolites were linked to dietary protein intake, with ten also associated with CKD progression (animal protein: 1, dairy: 7, red and processed meat: 2, nuts and beans: 1). These included amino acids, lipids, nucleotides, and other compounds. Notably, GPE (P-16:0/18:1), linked to red and processed meat, was associated with an 88% higher CKD risk, while 3- ureidopropionate showed a 48% lower risk.	Distinct metabolic properties and biomarkers of dietary protein sources suggest that metabolites form animal proteins, especially red and processed meat, may negatively impact kidney health in children. This highlights the need for protein source consideration in pediatric dietary guidelines.	Ren et al. [32]
Serum	A randomized clinical trial examined dietary protein restriction in CKD patients (age 18–70). Participants with moderate CKD (*n* = 585) followed either a moderate– or low-protein diet, while those with severe CKD (*n* = 255) followed a low- or very-low diet. Multivariable linear regression was used to analyze differences in log-transformed metabolite levels based on randomly assigned dietary protein intervention groups.	Untargeted metabolomic analysis/ RP-UHPLC/MS	130 metabolites differed significantly between participants on a low-protein vs. moderate-protein diet, and 32 metabolites between those on a very-low-protein diet vs. low-protein diet; 11 metabolites were consistently associated with protein intake across both studies: 3-methylhistidine, N-acetyl-3-methylhistidine, xanthurenate, isovalerylcarnitine, creatine, kynurenate, 1-(1-enyl-palmitoyl)-2-arachidonoyl-GPE (P-16:0/20:4), 1-(1-enyl-stearoyl)-2-arachidonoyl-GPE (P-18:0/20:4), 1-(1-enyl-palmitoyl)-2-arachidonoyl-GPC (P-16:0/20:4), sulfate, and γ-glutamylalanine.	Metabolomic blood biomarkers offer a valuable tool for assessing protein intake, aiding dietary interventions for CKD management. Given the challenges of long-term dietary modifications, metabolite measurements can help monitor adherence in clinical trials and guide targeted nutrition counseling for improved patient outcomes.	Rebholz et al. [33]
Kidney tissue	A study on mice with adenine-induced chronic kidney disease divided into three groups (*n* = 10 per group): (1) control group—fed a standard diet, (2) model group—fed a diet containing 0.2% adenine; and (3) sheep milk group—fed a diet with 0.2% adenine supplemented with 1.25 mL of sheep milk fed for four weeks.	Proteomic analysis/SDS-PAGE and LC-MS/MS Non-targeted metabolomic analysis/LC-MS/MS	Proteomic analysis revealed decresed expression of fibrosis-associated proteins, including VCAM1 and collagen, suggesting a role in slowing CKD progression. Metabolomic profiling showed decresed TMAO levels, a biomarker of kidney damage. Integrated data confirmed the down-regulation of the JAK1/STAT3/HIF-1α signaling pathway, contributing to the delay in renal injury.	These findings suggest that sheep milk may protect against kidney damage in CKD by inhibiting fibrosis and inflammation through the JAK1/STAT3/HIF-1α pathway. Its anti-inflammatory, antifibrotic, and anti-apoptotic properties make it a potential adjunct for CKD management. However, further research is needed to identify the specific bioactive compounds responsible for these effects.	Wei et al. [35]

GC-MS—gas chromatography–mass spectrometry; LC-MS—liquid chromatography–mass spectrometry; RP-UHPLC/MS—reverse-phase ultra-performance liquid chromatography–tandem mass spectrometry; SDS-PAGE—sodium dodecyl sulfate–polyacrylamide gel electrophoresis; LC-MS/MS—liquid chromatography with tandem mass spectrometry, CKD—chronic kidney disease; GPE (P-16:0/18:1)—1-(1-enyl-palmitoyl)-2-oleoyl-glycerophosphoethanolamine; and TMAO—trimethylamine N-oxide.

**Table 2:** Summary of molecular signatures identified to date that are associated with high-fat diet (HFD) or Western diet (WD) intake and their potential links to chronic kidney disease (CKD).

Sample Type	Study Design	Type of Analysis/Analysis Platform	Major Findings	Reference
Kidney tissue	A study on 6-week-old male C57BL/6N mice divided into two groups (*n* = 7 per group): (1) control group—fed a normal chow diet (10% fat), (2) HFD group—fed a high-fat-diet (60% fat) for fourteen weeks.	Proteomic analysis/nanoHPLC MS/MS	The HF diet up-regulated renal proteins involved in lipid transport, storage, and localization. The HF diet altered lipid metabolism from peroxisomes to mitochondria by down-regulating peroxisomal proteins and key components of the PPAR pathway. Increased expression of HMGCS2, CPT2, FABP4, ACAA2, ACOT2, CPT1A, and ALDH2, while BDH1 and ACADL were down-regulated.	Dozio et al. [46]
Kidney tissue	A study on 10-week-old male Swiss Webster mice divided into four groups (*n* = 6 per group): (1) STD group—fed a standard diet, (2) SFA group—fed a diet rich in saturated fatty acids (3) HR group—fed a HFD diet rich in polyunsaturated fatty acids with a linoleic to α-linolenic acid ratio of 14:1 (4) LR group—fed a HFD diet rich in polyunsaturated fatty acids with a linoleic to α-linolenic acid ratio of 5:1 for three months.	Proteomic analysis/ 2-DE MALDI-TOF MS	SFA diet affected 11 proteins: 7 up-regulated (PRDX6, PRDX1, PPIA, LDHD, ACOT2, HIBADH, and ALDH6A1) and 4 down-regulated (HSPD1, Apo-E, IDH1, and ATP5F1B). In the HR group, 7 proteins were altered: 4 down-regulated (HSPA5, PRDX6, ATP5F1B, and ALDH6A1), and 3 up-regulated (PPIA, AKR1A1, and IDH2). The LR diet altered 12 proteins: 9 up-regulated (PRDX1, P4HB, AKR1A1, ENO1, ETHE1, IDH1, ETFA, ALDH6A1, and HAAO) and 3 down-regulated (IDH1, ATP5F1B).	Wypych et al. [47]
Kidney tissue	A study on 5-week-old male C57BL/6J mice divided into four groups: (1) control—standard chow diet (2) WD group—high-fat (42% kcal), high-saturated fatty acids (>60% total), and high-sucrose (34%) diet for 8 weeks, (3) control + AA—standard chow with aristolochic acid (AA) every three days for 3 weeks, and (4) WD + AA—WD diet with AA every three days for 3 weeks.	Proteomic analysis/ TMT-labeled peptides were analyzed on an Orbitrap Eclipse Tribrid Mass Spectrometer	Approximately 1000 proteins were differentially expressed in the kidneys of AA-treated mice on a WD compared to controls. WD exacerbated AA-induced down-regulation of carbon metabolism pathways, including glycolysis, pyruvate, TCA, and fatty acid metabolism, indicating impaired kidney energy homeostasis. This group also showed increased immune-related proteins, suggesting kidney inflammation. The WD diet alone altered 19 proteins compared to controls, with 12 expressed in the proximal tubules. Up-regulated proteins: Arg2, Ces1d, Glud1, Pck1, Ugt2b37, and Vill. Down-regulated proteins: Acmsd, Acsm3, Car3, Glul, Hsd11b1, and Phgdh.	Oe et al. [49]
Kidney tissue, serum	A study on 8-week-old male C57BL/6 mice divided into three groups (*n* = 7 per group): (1) control group—fed a standard diet, (2) HGD group—fed high-glucose diet (75.9% carbohydrate, 14.7% protein and 9.4% fat), (3) HFD group—fed a high-fat diet (25% carbohydrate, 15% protein and 60% fat)	Untargeted metabolomic analysis/ GC-MS	The HFD diet affected 28 metabolites (AA, FA derivatives and others), with 9 increased and 1 decreased in serum, and 6 in the kidney. These metabolites are involved in many metabolic pathways related to energy, amino acid, and lipid metabolism.	Xie et al. [50]
Serum, liver, muscle tissue	A study on male Sprague Dawley rats. Rats underwent 5/6 nephrectomy for CKD induction or sham surgery. After two weeks, rats were fed a standard chow diet (SCD) or a high-fat diet (HFD) for 16 weeks to produce rat models for CKD-induced insulin resistance or HFD.	Untargeted metabolomic analysis/ UPLC-MS/OPLS-DA	A total of 101 metabolites in serum, 59 in liver, and 41 in muscle were associated with CKD-induced IR, while 58 in serum, 38 in liver, and 17 in muscle were linked to HFD-induced IR. CKD affected tryptophan and arginine metabolism, whereas HFD impaired lipid and purine metabolism.	Xu et al. [51]

nanoHPLC/MS—nano-high-performance liquid chromatography–mass spectrometer; HMGCS2—3-hydroxy-3-methylglutaryl-CoA synthase 2 (mitochondrial); CPT2—carnitine palmitoyltransferase 2; FABP4—fatty acid binding protein 4; ACAA2—acetyl-CoA acyltransferase 2; ACOT2—acyl-CoA thioesterase 2; CPT1A—carnitine palmitoyltransferase 1A; ALDH2—aldehyde dehydrogenase 2; BDH1—3-hydroxybutyrate dehydrogenase 1; ACADL—acyl-CoA dehydrogenase long chain; 2-DE—two-dimensional gel electrophoresis; MALDI-TOF—matrix-assisted laser desorption and ionization (time-of-flight) mass spectrometry; PRDX6—peroxiredoxin-6; PRDX1—peroxiredoxin-1; PPIA—peptidyl-prolyl cis-trans isomerase A; LDHD—probable D-lactate dehydrogenase; ACOT2—acyl-coenzyme A thioesterase 2; HIBADH—3-hydroxyisobutyrate dehydrogenase; ALDH6A1—methylmalonate-semialdehyde/malonate-semialdehyde dehydrogenase [acylating]; HSPD1—60 kDa heat shock protein; Apo-E—apolipoprotein E; IDH1—isocitrate dehydrogenase [NADP] cytoplasmic; ATP5F1B—ATP synthase subunit beta; P4HB—prolyl 4-hydroxylase subunit beta; AKR1A1—aldo-keto reductase family 1 member A1; ENO1—alpha-enolase; ETHE1—persulfide dioxygenase; ETFA—electron transfer flavoprotein subunit alpha, mitochondrial; HAAO—3-hydroxyanthranilate 3,4-dioxygenase; Arg2—arginine 2; Ces1d—carboxylesterase 1d; Glud1—glutamate dehydrogenase 1; Pck1—phosphoenolpyruvate carboxykinase 1; Ugt2b37—UDP-glucuronosyltransferase; Vill—villin-like protein; Acmsd—aminocarboxymuconate semialdehyde decarboxylase; Acsm3—acyl-CoA synthetase medium chain family member 3; Car3—Protein C2-domain ABA-related 3; Glul—glutamine synthetase; Hsd11b1—11-β-hydroxysteroid dehydrogenase 1; Phgdh—D-3-phosphoglycerate dehydrogenase; GC-MS—gas chromatography–mass spectrometry; AA—arachidonic acid; FA—fatty acid; CKD—chronic kidney disease; HFD—high-fat diet; IR—insulin resistance; UPLC-MS/MS—ultra-performance liquid chromatography–tandem mass spectrometry; and OPLS-DA—orthogonal partial least square-discriminant analysis.

**Table 3:** Summary of molecular signatures identified to date that are associated with prebiotics intake and their potential links to chronic kidney disease (CKD).

Sample Type	Study Design	Type of Analysis/Analysis Platform	Major Findings	Reference
Cecal contents	A study on 10-week-old male Sprague Dawley rats (*n* = 9 per group) induced CKD with a diet containing 0.7% adenine for 2 weeks, followed by a three-week intervention with either digestible starch (amylopectin)—CKD-DS group—or indigestible starch (HAMRS2)—CKD-RS group. Both isocaloric diets contained 14.5% protein, 66.9% carbohydrate, and 18.6% fat.	Proteomic analysis/TMT—Orbitrap Fusion Tribrid mass spectrometer iBAQ HPLC	A total of 9386 unique proteins were identified, with 5834 quantified. In CKD-RS vs. CKD-DS rats, 125 proteins with reduced expression (enzymes, proteins associated with humoral immune response, epithelial–mesenchymal transition (thioredoxin, S100-A6)) while 54 increased (enzymes, immunoglobulins, annexins, ion channel proteins, and sodium pump proteins).	Zybailov et al. [60]
Plasma, urine	A nonrandomized, open-label, 3-phase crossover study with repeated measures. Of 17 eligible subjects, 13 completed treatment. Phases included pretreatment (weeks 1–8), p-inulin treatment (weeks 9–20, 8g p-inulin twice daily), and post-treatment (weeks 21–28).	Untargeted metabolomics analysis/ GC-MS	Urinary levels of carbohydrate metabolites, including raffinose, 1-kestose, and beta-gentiobiosis, increased. During p-inulin supplementation, urine levels of beta-sitosterol and 4-methylcatechol increased, with 4-methylcatechol, inversely correlated with p-cresol.	Sohn et al. [61]

TMT—tandem mass tag; iBAQ—absolute intensity-based quantification; HPLC—high-performance liquid chromatography; HAMRS2—high amylose maize-resistant start; CKD—chronic kidney disease; and GC-MS—gas chromatography–mass spectrometry.
